# A Plasma-Functionalized ECM Platform for Intraoral Inflammation Control: Comparative Effects of Hyaluronic Acid and N-Acetyl-L-Cysteine on Oral Keratinocyte Response

**DOI:** 10.3390/polym18080977

**Published:** 2026-04-17

**Authors:** Pedro U. Muñoz-González, Pascale Chevallier, Leyla Desparois, Sylvie Louise Avon, Fatiha Chandad, Diego Mantovani, Vanessa P. Houde

**Affiliations:** 1 Laboratory for Biomaterials and Bioengineering (LBB), Université Laval, Québec, QC G1V 0A6, Canada; pumug@ulaval.ca (P.U.M.-G.); pascale.chevallier@crchudequebec.ulaval.ca (P.C.); diego.mantovani@gmn.ulaval.ca (D.M.); 2 Oral Ecology Research Group (GREB), Faculty of Dental Medicine, Université Laval, Québec, QC G1V 0A6, Canada; leyla.desparois.1@ulaval.ca (L.D.); fatiha.chandad@greb.ulaval.ca (F.C.); 3 Faculty of Dental Medicine, Université Laval, Québec, QC G1V 0A6, Canada; sylvie-louise.avon@fmd.ulaval.ca

**Keywords:** hyaluronic acid, decellularized extracellular matrix, plasma functionalization, inflammation modulation, gingival keratinocytes, N-acetyl-L-cysteine

## Abstract

Oral mucosal ulcers sustain a persistent inflammatory and oxidative microenvironment that interferes with epithelial repair and delays healing. Although hyaluronic acid (HA) is used in oral wound management due to its biocompatibility and hydrating properties, its biological activity is highly context-dependent and can be compromised under inflammatory conditions. In contrast, N-acetyl-L-cysteine (NAC) is a well-established antioxidant with documented anti-inflammatory effects, yet its rapid clearance limits its effectiveness when applied locally. In this study, the effects of HA and NAC, individually and in combination, on metabolic activity and inflammatory responses of TNF-α–stimulated human gingival keratinocytes were evaluated. In parallel, the individual immobilization of HA or NAC onto plasma-activated decellularized extracellular matrix (dECM) films was investigated as a materials-oriented approach for potential localized intraoral applications. NAC significantly attenuated TNF-α-induced IL-6 and IL-8 secretion, reducing both cytokines by approximately 99%, while preserving keratinocyte metabolic activity. HA displayed limited immunomodulatory effects. The combined HA + NAC condition did not improve the response compared with NAC alone. Plasma treatment enabled stable individual grafting of HA and NAC onto dECM films, and both functionalized surfaces retained chemical stability under saliva-like conditions. Collectively, these findings identify NAC as the most effective anti-inflammatory candidate under the tested cellular conditions and support plasma-functionalized dECM films as a feasible platform for future biological evaluation in intraoral applications.

## 1. Introduction

Oral mucosal ulcers are among the most common inflammatory lesions affecting the oral cavity and may arise from multiple etiologic factors, most commonly infectious, immune-mediated, traumatic, or neoplastic processes [1]. Clinically, they occur in a wide range of contexts, including aphthous stomatitis, systemic inflammatory diseases, chemotherapy- and radiotherapy-induced mucositis, and periodontal and peri-implant disorders [1,2]. Despite their diverse etiologies, these lesions share a common pathological hallmark: a persistent inflammatory microenvironment characterized by excessive production of pro-inflammatory cytokines, oxidative stress, and dysregulated extracellular matrix (ECM) remodeling, all of which impair epithelial repair and delay healing [3,4,5]. Current topical treatments remain largely palliative, focusing on pain relief and barrier protection rather than actively modulating the underlying inflammatory and redox imbalance that drives lesion chronicity [6]. Conventional management of oral mucosal ulcers relies on topical pastes, protective films, and pharmacological agents that provide symptomatic relief but are limited by a rapid washout by saliva, short residence time, and minimal impact on the inflammatory microenvironment [6,7,8].

Gingival keratinocytes play a central role in oral mucosal homeostasis and wound healing. Beyond their barrier function, these cells actively participate in immune signaling by secreting cytokines and chemokines, such as interleukin-6 (IL-6) and interleukin-8 (IL-8), particularly in response to inflammatory stimuli, including tumor necrosis factor-α (TNF-α) [3,9]. While transient cytokine production is essential for host defense and tissue repair, sustained activation disrupts epithelial migration, promotes excessive neutrophil recruitment, and enhances protease activity, including matrix metalloproteinases (MMPs) such as MMP-9, ultimately delaying re-epithelialization and compromising tissue integrity [10,11,12]. Therefore, biomaterial-based strategies that can attenuate epithelial inflammation while preserving cell viability represent a promising therapeutic avenue for the management of intraoral wounds. Because oral mucosal ulcers are continuously exposed to saliva and mechanical irritation, local biomaterial-based systems have been proposed to improve drug retention and protect the lesion surface. Thin films, adhesive dressings, and hydrogel-based local platforms have been investigated as strategies to provide mechanical coverage, maintain a hydrated interface, and prolong the residence time of therapeutic agents at the wound site [2,6]. In this context, thin biomaterial films are especially attractive because they can adhere to the ulcer surface while simultaneously acting as protective and potentially bioactive interfaces. Ideally, such systems should combine a local barrier function with the capacity to modulate the inflammatory microenvironment.

Hyaluronic acid (HA) has been extensively explored for oral and dermatological applications due to its biocompatibility, hygroscopic nature, and role in tissue hydration and lubrication [13,14,15]. High-molecular-weight HA (HMW-HA) has been frequently described as anti-inflammatory, with reported immunomodulatory effects in macrophages and fibroblasts [16]. Consequently, HA-based gels, membranes, and coatings are commonly used in the management of oral ulcers and periodontal therapy [2]. However, accumulating evidence suggests that HA’s biological activity is highly context-dependent and strongly influenced by its molecular stability. Under inflammatory conditions, HA is susceptible to enzymatic degradation by hyaluronidases and to oxidative fragmentation by reactive oxygen species (ROS), generating low-molecular-weight fragments that can act as damage-associated molecular patterns (DAMPs) and activate pro-inflammatory signaling through Toll-like receptors and CD44 [14,15,17,18,19]. In epithelial systems, including keratinocytes, these processes may negate or even reverse the expected anti-inflammatory effects of exogenously applied HA, raising questions about its reliability as a standalone bioactive agent in inflamed oral tissues. This context-dependent instability underscores the need for complementary strategies to preserve HA’s function and enhance its therapeutic consistency [18,19].

In contrast, N-acetyl-L-cysteine (NAC) is a well-established antioxidant and redox-modulating molecule with a long history of clinical use. NAC serves as a precursor for glutathione synthesis, directly scavenges reactive oxygen species (ROS), and inhibits redox-sensitive inflammatory pathways, including NF-κB signaling [20,21,22]. These properties have positioned NAC as a promising therapeutic candidate for inflammatory and oxidative stress-driven pathologies, including respiratory diseases, chronic pain, and epithelial injury [10,20,21]. NAC has the potential to simultaneously suppress cytokine overproduction, reduce protease activity, and protect ECM components from oxidative degradation [23]. However, its rapid diffusion and clearance in the oral cavity limit its effectiveness when delivered in soluble form, underscoring the need for localized and sustained delivery strategies [24]. When incorporated into biomaterial platforms, NAC could simultaneously attenuate epithelial inflammation and reduce oxidative degradation of matrix-associated components, providing a clear rationale for its combination with HA or ECM-based dressings [25].

Decellularized extracellular matrix (dECM) materials have emerged as attractive platforms for tissue repair due to their inherent bioactivity, structural similarity to native tissues, and capacity to support cell adhesion and migration [26,27]. When derived from appropriate sources and processed under controlled conditions, dECM retains key biochemical cues while minimizing immunogenicity. When processed as thin films, dECM materials can function as bioactive wound dressings that mimic the native basement membrane while mechanically covering the ulcer. However, unmodified dECM cannot actively regulate inflammation or oxidative stress in the oral environment, motivating efforts to functionalize its surface with bioactive molecules [28]. Accordingly, surface functionalization approaches are required to confer active biological modulation without altering the bulk properties of the dECM film. Plasma treatment is a versatile, solvent-free approach that enables modification of only the first few atomic layers without altering the bulk properties of the material. Furthermore, plasma treatment using reactive gas mixtures such as N_2_, N_2_/H_2_, O_2_, or air can introduce functional groups onto the surface of biomaterials, thereby enabling the immobilization of therapeutic agents [29,30,31]. In the present study, an N_2_/H_2_ plasma mixture was selected to activate the dECM surface before HA or NAC immobilization. Although interest in plasma-modified ECM-based materials has increased, few studies have rigorously examined the chemical stability of immobilized bioactive molecules under highly degradative and physiologically relevant environments such as the oral cavity. In addition, despite the recognized relevance of HA and NAC in inflammatory and biomaterial-related applications, an important gap remains regarding their comparative performance in epithelial oral inflammation models and their suitability for incorporation into localized dECM-based platforms. In particular, it remains unclear which of these molecules provides the most relevant anti-inflammatory effect in TNF-α-stimulated gingival keratinocytes, and whether such candidate molecules can be individually immobilized onto plasma-activated dECM films while maintaining chemical stability under saliva-like conditions. Addressing this gap is important for the rational design of localized biomaterial systems for intraoral ulcer management.

**Figure 1:**
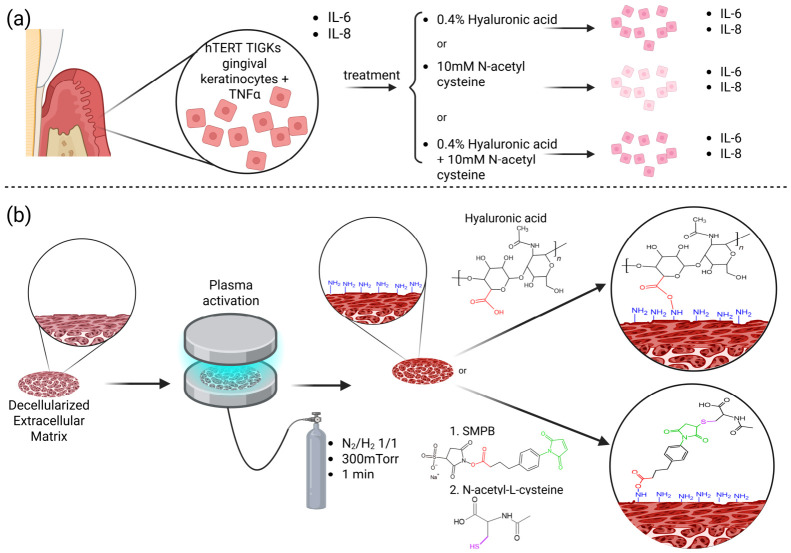
Schematic diagram that summarizes the two main stages of this paper, illustrating (**a**) the evaluation of the anti-inflammatory effect of hyaluronic acid and N-acetyl-L-cysteine, alone or in combination, on TNF-α-stimulated gingival keratinocytes, and (**b**) the process for the functionalization of decellularized extracellular matrix with hyaluronic acid or N-acetyl-L-cysteine, via plasma activation.

In this study, we comparatively evaluated the effects of HA and NAC, alone and in combination, on the metabolic activity and inflammatory response of TNF-α-stimulated gingival keratinocytes (Figure 1a). This cellular analysis was intended to identify which molecule showed the most relevant anti-inflammatory profile under the selected epithelial inflammatory conditions. In parallel, we assessed the feasibility and chemical stability of immobilizing HA or NAC individually onto plasma-activated dECM films under saliva-like conditions (Figure 1b), as a first step toward the development of localized intraoral biomaterial surfaces. Rather than demonstrating the biological performance of functionalized films, this work aims to clarify the relative contributions of HA and NAC in an epithelial inflammatory context and to examine plasma-functionalized dECM films as a material platform for future localized intraoral applications.

## 2. Materials and Methods

### 2.1. Effect of the Stimulation of HA and NAC on the Metabolic Activity of Gingival Keratinocytes

Gingival keratinocytes hTERT TIGKs cells (ATCC CRL-3397, Manassas, VA, USA) were cultivated in Keratinocytes Serum-Free Medium (SFM) (Life Technologies, Carlsbad, CA, USA) supplemented with 100 μg/mL penicillin/streptomycin + 0.25 μg/mL amphotericin B at 37 °C in 5% CO_2_. In total, 5 × 10^3^ cells/well were seeded in 96-well plates and incubated for 24 h at 37 °C in 5% CO_2_. The cells were pre-treated for 2 h with 0.4% (*m*/*v*) high molecular weight (HMW) hyaluronic acid (HA) (R&D Systems, Toronto, ON, Canada), 10 mM N-acetyl-L-cysteine (NAC) (Sigma-Aldrich, Oakville, ON, Canada) (pH adjusted to 7.5) or the combination of both prior being stimulated for 24 h with 10 ng/mL of human recombinant TNF-α (Life Technologies). Untreated or unstimulated cells were used as controls. After 24 h, the hTERT TIGKs metabolic activity was quantified by using an MTT (3-[4,5-dimethylthiazol-2-yl]-2,5 diphenyl tetrazolium bromide) colorimetric assay (Sigma-Aldrich) according to the manufacturer’s protocol.

### 2.2. Quantification of the Inflammatory Molecules Production by Gingival Keratinocytes in Response to HA and NAC Stimulation

hTERT TIGKs cells were cultivated in Keratinocytes SFM supplemented with 100 μg/mL penicillin/streptomycin + 0.25 μg/mL amphotericin B at 37 °C in 5% CO_2_. Cells were seeded in 12-well plates and incubated at 37 °C in 5% CO_2_ until they reached 90% confluence. The cells were then pre-treated for 2 h with 0.4% (*w*/*v*) HMW-HA, 10 mM NAC (pH adjusted to 7.5), or the combination of both prior to being stimulated for 24 h with 10 ng/mL of human recombinant TNF-α, as described above. Untreated or unstimulated cells were used as controls.

#### 2.2.1. Pro-Inflammatory Cytokines Measurement

After 24 h of treatment and stimulation, the cell supernatants were collected, and pro- inflammatory cytokines (IL-6, IL-8) and matrix metalloproteinases (MMP-9) were then measured by ELISA according to the manufacturer’s protocols (R&D Systems). The cells were washed, lysed, and the proteins were quantified by the BCA method (Pierce, Thermo Fisher, Mississauga, ON, Canada), to normalize the results, which are expressed as pg/mL/μg of protein.

#### 2.2.2. Mitochondrial Superoxide Measurement by Flow Cytometry

After 24 h of treatment and stimulation, the cells were washed with warm PBS (37 °C), scraped, and stained with 1 μM of MitoSox Red dye (Life Technologies) according to the manufacturer’s protocol. Mitochondrial superoxide levels were then assessed by flow cytometry on a Cytek Northern Lights (Cytek Biosciences, Fremont, CA, USA), and analyzed with FlowJo (BD Biosciences, Mississauga, ON, CA).

### 2.3. Decellularized Extracellular Matrix Films Preparation

The production of the decellularized extracellular matrix films was carried out from decellularized bovine pericardium, generously supplied by Tissuegraft Srl (Novara, Italy) (patent number 102020000007567, submitted on 9 April 2020). The films were fabricated using a standard solvent-casting approach. In brief, the decellularized pericardial tissue was first enzymatically digested and subsequently lyophilized before film formation. Next, 1.2 g of the resulting lyophilized dECM was dissolved in 100 mL of 0.05 M acetic acid under continuous stirring. A 4 mL aliquot of this solution was then cast into a 37 mm diameter Teflon mold, and the solvent was allowed to evaporate at room temperature in a custom-made chamber with a dry-air flow for 12 h. The dried films were subsequently rinsed three times with deionized water and left to dry overnight at room temperature. The resulting films were easily removable from the mold and had an approximate thickness of 50 μm.

### 2.4. Plasma Activation of the dECM Materials and Their Functionalization with HA or NAC

The dECM films were modified by a microwave-sourced low-pressure plasma system (Plasmionique Inc., Sainte-Julie, QC, Canada), as described previously [31]. Briefly, the films were treated under discharge with a mix of N_2_/H_2_ (1/1) at 300 mTorr for 1 min. Then, the resulting aminated dECM films were directly used for further grafting with HA or NAC. Because HA and NAC required different coupling strategies under the selected experimental conditions, simultaneous HA + NAC grafting onto plasma-activated dECM was not performed.

#### 2.4.1. HA Grafting

HA was dissolved in (2-(N-morpholino)ethanesulfonic acid) buffer (MES) (pH 4.75) to get a final concentration of 0.4% *m*/*v*. Then, the carboxylic groups of HA were activated with 3 mg/mL of (1-ethyl-3-(3-dimethylaminopropyl) carbodiimide hydrochloride) (EDC) added every 10 min, three times, until the reaction was complete (30 min). The activated HA solution was poured onto the previously plasma-aminated dECM films and left to react for 2 h to allow a covalent bond to be formed. Thereafter, the grafted samples were washed five times with deionized water and air dried.

#### 2.4.2. NAC Grafting

The plasma-aminated dECM films were reacted with a 3 mg/mL solution of sulfo-succinimidyl-4-(p-maleimidophenyl)butyrate (SMPB) in PBS (pH 7.4) for 1 h under light protection. After washing three times with deionized water, the SMPB-grafted dECM films were further left react with a solution of NAC at 10 mM in PBS buffer (pH 7.4) for 2 h. Thereafter, the grafted samples were washed five times with deionized water and air dried. Regarding the nature of the functionalization protocols for both molecules, it was not possible to perform the combined HA + NAC functionalization.

### 2.5. Effect of the Incubation of the Functionalized dECM in a Saliva-like Medium on Their Stability

Grafted samples and an unmodified control were sterilized by UV (254 nm) with two cycles of 15 min with a pause of 5 min between both (to avoid sample degradation/heating). Then, they were incubated with sterile pseudo-saliva medium in an incubator (37 °C, 5% CO_2_) for 7 days [32]. After washing five times with deionized water and air drying, the samples were characterized.

### 2.6. Surface Characterization

X-ray photoelectron spectroscopy (XPS): The surface atomic chemical composition of the dECM untreated and modified was analyzed by XPS using a PHI 5600-ci equipment (Physical Electronics, Chanhassen, MN, USA) at a collecting angle of 45° with respect to the normal surface. A standard Al Kα (1486.6 eV) X-ray source was used to record the survey spectra with charge neutralization, whereas high resolution (HR) spectra of C1s and O1s were recorded without. The analyzed area was 500 µm^2^. Triplicate samples for each condition were analyzed. The curve fitting was performed using a Gaussian–Lorentzian peak fitting procedure by least squares, after subtracting the background according to the Shirley method. The C1 peaks were set at 285 eV (C-C and C-H) as a reference.

#### Contact Angle Measurements

Static water contact angle (WCA) measurements were performed on dECM films using a VCA Optima XE (AST Products, Billerica, MA, USA) instrument. The measurements were performed with a drop of deionized water of 0.5 μL. For each film, at least five measurements at different locations were averaged, and three replicates per condition were tested.

### 2.7. Statistical Analysis

All the experiments were independently performed at least in triplicate, repeated three times. The mean and the standard deviation (S.D.) are presented for each data set. Data sets were compared using analysis of variance (ANOVA). The difference in means was assessed using a Fisher post hoc test and was considered statistically significant at the *p* < 0.05 level.

## 3. Results

### 3.1. Metabolic Activity of Gingival Keratinocytes Stimulated with Hyaluronic Acid and N-Acetyl-L-Cysteine

To assess the cytotoxic effect of HA and NAC, hTERT TIGKs gingival keratinocytes were stimulated with different concentrations of HMW-HA and NAC, either alone or in combination. No significant differences (*p* < 0.05) were detected between 0.2% and 0.4% HA groups (HA0.2 and HA0.4, respectively) and the control. In contrast, NAC at 5 and 10 mM (NAC5 and NAC10, respectively) significantly increased keratinocytes metabolic activity compared to the control. When combined, all HA–NAC groups showed significantly higher metabolic activity than the control. Although no significant differences (*p* < 0.05) were observed among the groups, a tendency to increase metabolic activity was noted due to a higher amount of NAC (Figure S1). Based on these results, the highest concentrations, 0.4% HA and 10 mM NAC, were selected for subsequent experiments.

### 3.2. Production of Inflammation-Related Molecules by Gingival Keratinocytes Stimulated by Hyaluronic Acid and N-Acetyl-L-Cysteine

To evaluate the anti-inflammatory effect of hyaluronic acid and N-acetyl-L-cysteine on pro-inflammatory gingival keratinocytes, inflammatory cytokines IL-6 and IL-8, and MMP-9, were quantified from the culture medium of TNFα-stimulated keratinocytes treated with HA and/or NAC.

**Figure 2:**
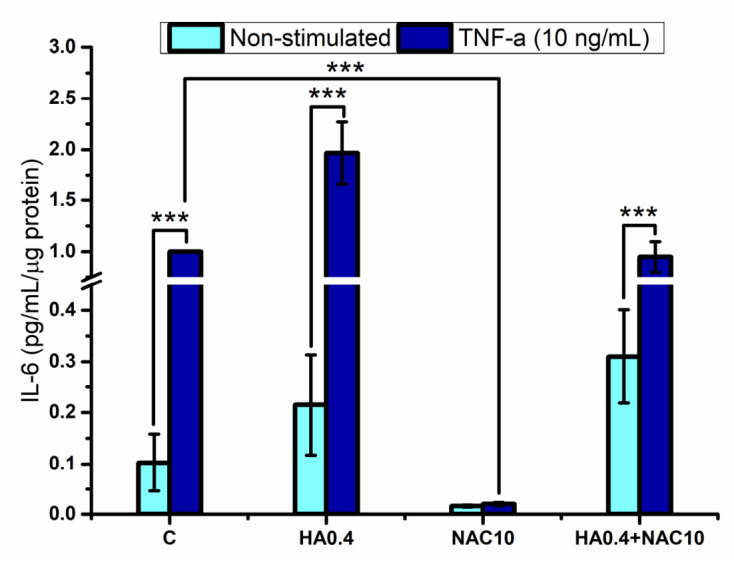
Interleukin-6 produced by the gingival keratinocytes stimulated by hyaluronic acid and/or N-acetyl-L-cysteine. Legend: C refers to unstimulated keratinocytes control, HA0.4, NAC10, HA0.4 + NAC10 refer to keratinocytes stimulated with 0.4% HA, or 10 mM NAC, and both together, respectively. Results are reported as normalized data mean (large bar *n* = 3), ±standard deviation (short bars). *** is referred to as statistically significant differences between the marked groups, ANOVA–Fisher (*p* < 0.001, respectively).

As shown in Figure 2, when keratinocytes are stimulated with TNF-α, they secrete a significantly larger amount of the proinflammatory cytokine IL-6 in the control, HA0.4, and HA0.4 + NAC10 groups. In the presence of NAC, significantly less IL-6 was produced than in the other groups; no significant differences (*p* < 0.05) were detected between the unstimulated and TNF-α-stimulated cell cultures either.

**Figure 3:**
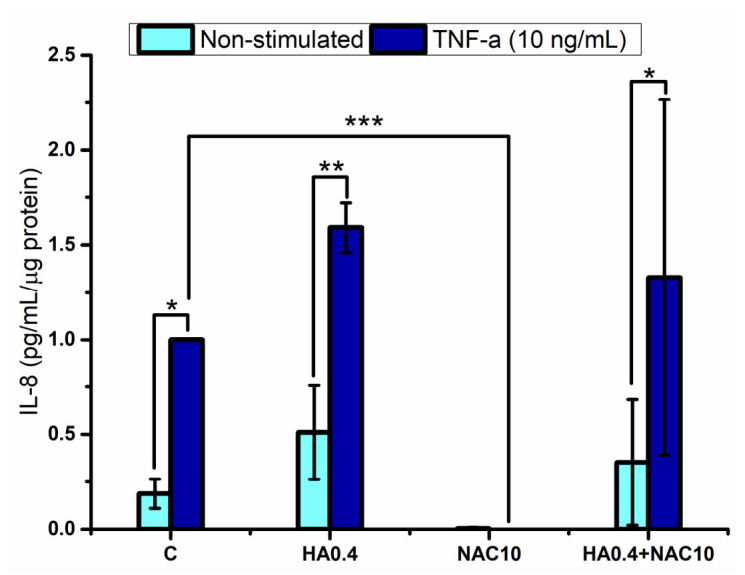
Interleukin-8 produced by the gingival keratinocytes stimulated by hyaluronic acid and/or N-acetyl-L-cysteine. Legend: C refers to unstimulated keratinocytes control, HA0.4, NAC10, HA0.4 + NAC10 refer to keratinocytes stimulated with 0.4% HA, or 10 mM NAC, and both together, respectively. Results are reported as normalized data mean (large bar *n* = 3), ±standard deviation (short bars). *, **, *** are referred to as statistically significant differences between the marked groups, ANOVA–Fisher (*p* < 0.05, 0.01, 0.001, respectively).

A similar pattern was observed for the pro-inflammatory cytokine IL-8 (Figure 3). When keratinocytes were stimulated with TNF-α, they secreted significantly more IL-8 in the control, HA0.4, and HA0.4 + NAC10 groups. In the presence of NAC, significantly less IL-8 was produced than in the other groups; no significant differences (*p* < 0.05) were detected between the unstimulated and TNF-α-stimulated cell cultures either. The increase in the production of IL-6 and IL-8 by the HA0.4-containing groups may be associated with a possible degradation of the hyaluronic acid into smaller fragments, which have been reported to induce a pro-inflammatory response [16].

**Figure 4:**
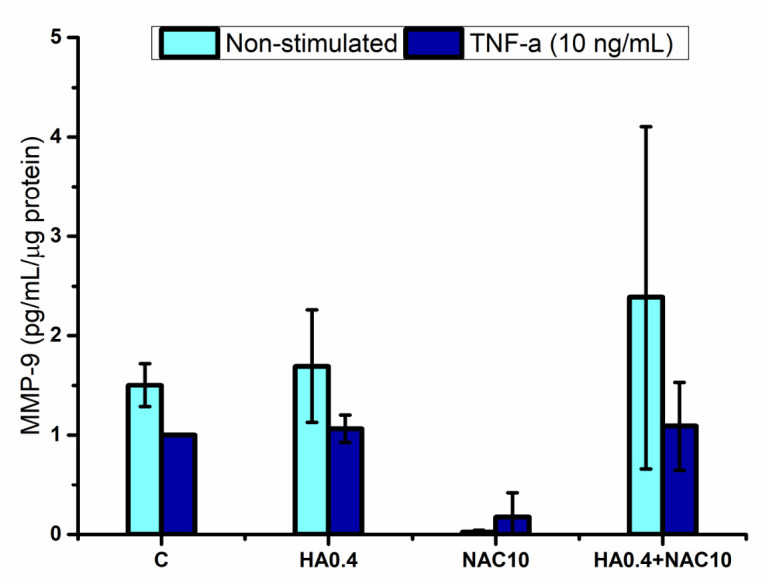
Matrix metalloproteinase 9 produced by the gingival keratinocytes stimulated by hyaluronic acid and/or N-acetyl-L-cysteine. Legend: C refers to unstimulated keratinocytes control, HA0.4, NAC10, HA0.4 + NAC10 refer to keratinocytes stimulated with 0.4% HA, or 10 mM NAC, and both together, respectively. Results are reported as normalized data mean (large bar *n* = 3), ±standard deviation (short bars). No statistically significant differences were detected, ANOVA–Fisher (*p* < 0.05).

MMP-9 levels did not show a significant increase following TNF-α stimulation under the experimental conditions used (Figure 4). In fact, no statistically significant differences were detected (*p* < 0.05) among the evaluated groups, including HA-, NAC-, and HA + NAC-treated keratinocytes. Therefore, the MMP-9 results should be interpreted with caution, and no robust conclusion can be drawn regarding the modulatory effects of HA or NAC on this marker in the present model.

To evaluate the production of mitochondrial superoxide levels in the keratinocytes in the presence of the HA and NAC, median MitoSox coloration was measured by Flow cytometry. HA and NAC showed a tendency to decrease the mitochondrial superoxide production in gingival keratinocytes (Table 1).

### 3.3. dECM Modification, Characterization, and Stability

#### 3.3.1. Efficiency of the ECM Plasma Modification and Functionalization

Plasma activation was performed on the surface of the ECM films to enhance the amount of amine groups on the surface, to further facilitate the direct covalent bonding of HA or NAC through a linking arm (SMPB). This approach enabled the efficient individual grafting of each bioactive compound, although the simultaneous immobilization of HA and NAC could not be achieved. The efficiency of the modification processes was assessed by XPS survey analyses. The results are given in Table 2.

Plasma treatment with N_2_/H_2_ led to an increase in the relative N content to 9.7 ± 0.8% compared to the pristine dECM 6.3 ± 2.8%, as expected, while it did not influence the O/C ratio (Table 2). This means that the surface was successfully activated without altering the dECM composition itself. This functionalization, which leads to the formation of amino groups, enabled the molecules of interest, HA and NAC, to be covalently grafted onto the surface. Thanks to the carboxylic groups of HA, this molecule could be directly grafted onto the aminated surface (Figure 1b). After grafting, a slight increase in the amounts of C and O, the main elements of HA, was observed, while a significant decrease in the N/C ratio was observed. This may be associated with the successful grafting of hyaluronic acid molecules, hindering the N species of the substrate (XPS depth analysis ~5 nm). Due to the chemical structure of NAC, a linking arm, in this case SMPB, was used. In fact, the succinimidyl group of this linking arm reacted with the amino groups on the functionalized surface of dECM, resulting in a terminal maleimide group that then allowed the covalent bonding of NAC via its terminal thiol functionality (Figure 1b). After SMPB grafting, the amount of C increased while that of N decreased significantly compared to aECM, from 9.7 ± 0.8% to 1.2 ± 0.3% (Table 2). This resulted in a significant decrease in the N/C ratio compared to aECM, which is explained by the composition of SMPB. When NAC was subsequently grafted, both O and N amounts increased compared to the SMPB-grafted surface, and consequently, the O/C and N/C ratios also increased. Since NAC is a small molecule containing O and N in addition to C, these results tend to demonstrate the success of the NAC grafting.

**Figure 5:**
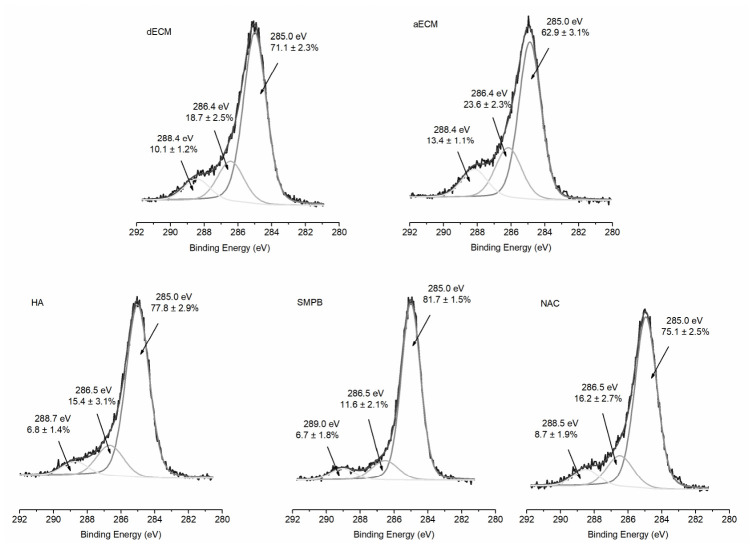
High-resolution XPS C1s analysis of the treated dECM samples, where the spectra were deconvoluted into three main peaks, each associated with C-C/C-H, C-O/C-N, and N-C=O or O-C=O bonds. Legend: dECM refers to pristine decellularized ECM; aECM refers to dECM plasma-activated; HA, SMPB, and NAC refer to aECM functionalized with HA, SMPB, and SMPB + NAC, respectively.

The efficiency of the surface modification and grafting was further investigated by high-resolution spectra (HR) of C1s and O1s. All HR C1s spectra were deconvoluted into three main peaks at 285.0, 286.4, and 288.1–289.0 eV be assigned to C-C/C-H, C-O/C-N, and N-C=O or O-C=O bonds, respectively [31,33]. After plasma modification, an increase in the contribution of the bands at 286.4 eV from 18.7 ± 2.5% to 23.6 ± 2.2%, and at 288.4 eV was observed (Figure 5). These results, combined with the increase in the N/C ratio (Table 2), confirm the formation of N-containing species, such as amines (C-N) and amides (NH-C=O), in accordance with the literature. Indeed, after nitrogen-rich plasma treatments on natural polymer films, such as collagen, cellulose, or chitosan, similar observations were made: an increase in nitrogen- and oxygen-containing functional groups [34,35]. After HA grafting, an increase in the C-C/C-H band, associated with glycosaminoglycan structure, was observed, balanced by a decrease in C-O/C-N and NH-C=O. Furthermore, in HR O1s, the increase in the peak at 533.3 eV from O-C, compared to the plasma-treated sample, clearly demonstrated the successful grafting of HA (Figure S2). Regarding the NAC grafting via SMPB as a linking arm, first, in the SMPB HR C1s spectrum compared to plasma treated one, the significant increase in C-C/C-H as well as the shift in the carbonyl band at 289.0 eV associated with maleimide groups confirmed the grafting of the linking arm. After NAC grafting, a decrease in C-C/C-H band was observed, compensated by an increase in C-O/C-N and C=O, which can be explained by the NAC chemical structure. In addition, a shift in the carbonyl band towards lower binding energies (from 289.0 eV to 288.4 eV) was observed, which corroborated the NAC grafting.

#### 3.3.2. Effect of the dECM Functionalization on Its Surface Wettability and Resistance to Degradation

The wettability of the surface was then evaluated, as this is a key parameter in this study. In fact, the goal of this work is to obtain a bioactive film to treat ulcers on the one hand, and on the other hand, to have a barrier effect to prevent the permeation of saliva. Furthermore, it is recognized in the literature that surface wettability will have an impact on the interactions of the biomaterial with its environment. The WCA from the raw dECM film decreased from 96.1 ± 1.5° to 86.5 ± 5.6° after N_2_/H_2_ plasma treatment. This increase in hydrophilicity was associated with the insertion of nitrogen- and oxygen-containing functional groups on the surface. In fact, the presence of such polar functional groups on polymeric surfaces is well known to play a key role in improving wettability [36]. It is interesting to note that plasma treatment did not affect the hydrophobicity of the other side of the film, which would prevent saliva permeation, as intended. After HA grafting, the WCA decreased from 86.5 ± 5.6° to 72.4 ± 2.4°, and this decrease can be attributed to the O-rich structure of HA. However, for NAC grafting, WCA remained 84.5 ± 3.5°, which could be explained by the presence of terminal acetylamino groups of NAC on the surface, as seen in XPS (peak at 288.5 eV).

**Figure 6:**
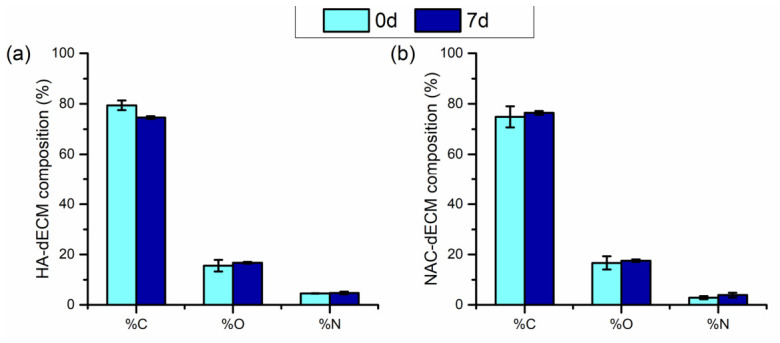
Relative atomic percentage, assessed by XPS analyses, of the (**a**) HA- and (**b**) NAC-ECM films grafted before and after immersion in saliva-like medium for 7 days. Legend: HA-, and NAC-dECM refers to dECM functionalized with HA and NAC, respectively. Results are reported as normalized data mean (large bar *n* = 3), ±standard deviation (short bars). No statistically significant differences were detected, ANOVA–Fisher (*p* < 0.05).

The samples were analyzed after seven days of treatment in a saliva-like medium to evaluate whether the medium had any effect on their composition. No differences were noticed (*p* < 0.05) regarding the chemical composition before and after the immersion test (Figure 6), either for HA or NAC. In addition, HR C1s spectra did not display a significant difference after the immersion test when compared to the “fresh” grafted sample. These results suggest that the dECM grafted either by hyaluronic acid or N-acetyl-L-cysteine was stable during that period of treatment.

## 4. Discussion

In this study, we evaluated whether hyaluronic acid and N-acetyl-L-cysteine can modulate inflammatory signaling in gingival keratinocytes and whether these molecules can be stably immobilized onto plasma-activated dECM films for potential intraoral therapeutic use. The results reveal a marked contrast between the biological efficacy of NAC and the limited, and in some contexts counterproductive, effects of high molecular weight HA.

HA maintained keratinocyte metabolic activity, consistent with prior literature reporting its general biocompatibility [13]. However, its biological contribution did not extend beyond preserving viability. NAC, in contrast, enhanced metabolic activity in a dose-dependent fashion, reflecting its antioxidant properties and its ability to stabilize mitochondrial function [37,38]. The HA + NAC combination provided no added benefit over NAC alone, suggesting that HA does not participate meaningfully in early cellular activation under inflammatory conditions. These findings indicate that, under the inflammatory conditions evaluated here, NAC was the main bioactive modulator, whereas HA contributed little to the suppression of pro-inflammatory signaling.

The inflammatory response further exposes the limitations of HA. TNF-α stimulation strongly increased IL-6 and IL-8 secretion [3,9], yet HA at 1500–1800 kDa failed to suppress pro-inflammatory cytokine secretion, contradicting the assumption that HMW-HA consistently exerts anti-inflammatory effects [16]. Multiple mechanistic factors may explain this discrepancy. Although HMW-HA has been reported to elicit anti-inflammatory effects in macrophages, including downregulation of iNOS, TNF-α, and IL-6 [16], keratinocyte responses appear more variable. Indeed, studies show that keratinocytes exposed to oxidative stimuli such as UV radiation reduce cytokine secretion regardless of HA molecular weight [39]. To contextualize these discrepancies, it is important to consider HA stability under inflammatory conditions. HA is highly susceptible to enzymatic degradation by hyaluronidases (HYALs), specifically by HYAL-1 and HYAL-2, and to non-enzymatic fragmentation by ROS [14,15,17], both of which increased markedly during inflammation. As a result, exogenous HMW-HA likely undergoes rapid conversion into low molecular weight fragments known to promote pro-inflammatory signaling and act as DAMPs via TLR2, TLR4, and CD44, promoting the activation of NF-κB, and then the production of pro-inflammatory cytokines IL-1β, TNF-α, and IL-8 (Figure S3) [16,18,19]. This destabilization fundamentally alters HA’s biological profile and likely accounts for the absence and potential reversal of anti-inflammatory activity in our keratinocyte model [14,15,16,17,39,40].

In contrast, NAC exerted a strong anti-inflammatory effect. IL-6 and IL-8 levels in NAC-treated keratinocytes returned to those of unstimulated controls. This aligns strongly with NAC’s established ability to replenish glutathione, scavenge ROS, and inhibit NF-κB activation [20,21]. Because mitochondrial superoxide contributes to both cytokine amplification and HA fragmentation, NAC’s redox-modulating activity targets two major drivers of epithelial inflammation [22]. Because in this study, mitochondrial superoxide measurements were based on only two independent samples, these observations show trends rather than conclusive evidence of oxidative modulation. The HA + NAC condition did not simply fail to improve the response compared with NAC alone but instead was associated with markedly higher IL-6 and IL-8 levels than NAC alone, suggesting a possible antagonistic or interfering interaction under the tested conditions, mainly caused by HA presence. The clinical relevance of suppressing IL-6 and IL-8 is substantial. IL-6 disrupts epithelial homeostasis and contributes to ulcer chronicity [4], whereas IL-8 promotes excessive neutrophil infiltration and ECM degradation [5]. NAC’s ability to neutralize both mediators positions it as a particularly strong candidate for managing oral ulcers characterized by persistent epithelial inflammation. MMP-9 did not show the expected increase after TNF-α stimulation in the present keratinocyte model. This result was unexpected, as MMP-9 is commonly associated with inflammation-related tissue remodeling and epithelial barrier disruption [41]. However, MMP-9 regulation in keratinocytes may depend strongly on the inflammatory context, including the nature and combination of cytokine stimuli, the duration of stimulation, baseline protease expression, and cell-specific responsiveness [42]. In this regard, previous studies have shown that TNF-α can induce MMP-9 expression in keratinocyte models. Still, the magnitude of this response appears to vary depending on the epithelial system, stimulation conditions, and inflammatory context [41,42]. Therefore, the current data does not support a definitive interpretation of HA- or NAC-mediated modulation of this marker, and MMP-9 should be considered an exploratory outcome in this study rather than a conclusive indicator of inflammatory regulation.

The characterization of the dECM materials’ surface composition supports the robustness of the immobilization strategy. Plasma activation increased the N/C ratio of dECM surfaces, reflecting the incorporation of nitrogen-rich functional groups and enabling subsequent HA or SMPB attachment through chemical bonding rather than physisorption, as it was previously reported [31]. Notably, both HA- and NAC-functionalized films retained their chemical composition after seven days in a saliva-like medium, demonstrating resistance to hydrolytic challenge. This temporal stability is essential, as the early windows of inflammatory modulation and epithelial migration occur primarily within the first 24–72 h of ulcer healing [1,2,6]. Although plasma functionalization of ECM-derived substrates has been explored, only a limited number of recent reports describe this approach, particularly in relation to chemical durability under physiologically relevant conditions. Nitrogen plasma treatment has been shown to incorporate nitrogen functionalities into collagen–polycaprolactone fibers, decreasing water contact angle from 130° to 52° and enhancing cell proliferation [29], while air plasma activation of ECM–polyvinyl alcohol composites introduces amide and carbonyl groups that support improved cell attachment [30]. In this study, plasma activation was employed specifically to enrich the surface with amine-reactive sites, enabling the immobilization of the bioactive molecules HA and NAC through the bonding with the SMPB linking arm. In the present system, simultaneous immobilization of HA and NAC onto plasma-activated dECM was not achieved under the selected experimental conditions, since they required different coupling strategies: HA was directly immobilized through carbodiimide-mediated activation of carboxyl groups, whereas NAC grafting required prior introduction of the maleimide-bearing linker SMPB to react through its thiol group. Successive functionalization steps on plasma-activated surfaces are challenging because the number of accessible NH_2_ groups is limited and may decrease over time due to surface reorganization. Moreover, the large size of HA can generate steric hindrance, reducing access to remaining reactive sites. To maximize its anti-inflammatory activity, NAC should ideally be grafted after HA so that it remains more accessible at the surface. However, simultaneous grafting of both molecules does not allow precise control of the proportion of each species attached to the surface. Since the combined HA + NAC stimulation did not provide a relevant advantage over NAC alone in the keratinocyte inflammatory model, additional optimization of simultaneous co-grafting was not pursued in the present study. Therefore, although both molecules could be individually immobilized, achieving dual functionalization will likely require further optimization of grafting sequence, linker design, and reaction conditions. Future work could evaluate whether HA should be immobilized first, followed by a second and separately optimized NAC grafting step through the linker-mediated route, or whether NAC could first be grafted onto HA in a controlled manner, followed by immobilization of the resulting NAC-HA conjugate onto dECM. The fact that both coatings remain chemically stable under saliva-like conditions represents an interesting outcome compared to the existing literature, where long-term retention of bioactive molecules on ECM surfaces is rarely characterized and often assumed rather than demonstrated. This stability directly strengthens the translational relevance of the platform, suggesting that the functionalized dECM films can withstand the highly degradative intraoral environment long enough to exert therapeutic benefit during the most critical phases of mucosal repair.

According to our biological assessments, NAC, rather than HA, was the main anti-inflammatory modulator under the soluble-cellular conditions evaluated in this study [21]. In parallel, plasma-activated dECM proved to be an effective platform for the individual immobilization of HA or NAC, and both functionalized surfaces remained chemically stable under saliva-like conditions. However, the biological performance of the functionalized dECM films themselves was not directly evaluated in the present work. Therefore, these materials should be regarded as promising platforms for future biological validation rather than as therapeutically validated surfaces. HA may still contribute to hydration, lubrication, and mechanical protection, but its biochemical role under inflammatory and oxidative conditions appears limited in the present keratinocyte model [16]. Since HA did not show a relevant advantage in the keratinocyte model and could not be simultaneously co-grafted with NAC under the selected conditions, its role should be regarded as a design constraint that requires further optimization, stabilization, or reformulation in future work. A limitation of the present study is that immunomodulatory effects were evaluated at the cellular level using soluble HA and NAC, while direct assessment of immune and inflammatory responses to HA- and/or NAC-functionalized dECM scaffolds remains to be performed. Future studies will therefore focus on quantifying NAC release kinetics, evaluating mechanical performance under dynamic conditions that mimic mastication, and assessing fibroblast and immune cell responses, as well as therapeutic efficacy in ex vivo or in vivo models of oral ulceration. Such investigations will be essential to determine whether the strong in vitro redox-modulating and cytokine-suppressing properties of NAC translate into clinically meaningful improvements in mucosal wound healing.

## 5. Conclusions

N-acetyl-L-cysteine effectively modulated the inflammatory response in TNF-α-stimulated gingival keratinocytes, reducing IL-6 and IL-8 secretion while preserving cell metabolic activity, whereas high-molecular-weight hyaluronic acid maintained viability but showed limited immunomodulatory effects under the tested conditions. The HA + NAC combination did not improve the response compared with NAC alone. Plasma-activated dECM films enabled individual immobilization of HA and NAC, and their surface elemental composition remained unchanged after exposure to a saliva-like medium as assessed by XPS, suggesting stability under simulated oral conditions. These findings identify NAC as the most promising anti-inflammatory candidate in the present soluble-cell model and support plasma-functionalized dECM films as a feasible material platform for future biological evaluation in localized oral applications. In contrast, the role of HA in the final platform remains less clearly defined, since its anti-inflammatory contribution was limited under the tested conditions, and its combination with NAC could not be implemented through simultaneous co-functionalization. However, although HA- and NAC-functionalized dECM films showed chemical stability under saliva-like conditions, their direct biological performance was not evaluated in the present study. Future work should therefore examine keratinocyte adhesion, viability, and inflammatory responses directly on the functionalized surfaces, together with their performance under dynamic oral-like conditions and in more complex models relevant to oral ulceration.

## Figures and Tables

**Table 1:** Mitochondrial superoxide levels in keratinocytes treated with HA and NAC, determined from median MitoSOX fluorescence intensity.

Group	Median of MITOSOX-Positive Cells
Unstained	8.024 ± 2.17
TNF-α	114.92 ± 77.46
TNF-α + HA0.4	62.77 ± 52.14
TNF-α + NAC10	35.36 ± 26.61
TNF-α + HA0.4 + NAC10	37.81 ± 28.99

TNF-α, +HA0.4, NAC10, +HA0.4 + NAC10 refer to keratinocytes treated with TNF-α alone, or additionally stimulated with HA0.4, NAC10, or both, respectively. Results are reported as data mean (*n* = 2), ±standard deviation.

**Table 2:** Relative surface concentration of C, O, and N, and their respective ratios.

Group	%C	%O	%N	O/C	N/C
dECM	80.8 ± 1.3	12.8 ± 2.7	6.3 ± 2.8	0.16 ± 0.03	0.08 ± 0.03
aECM	76.4 ± 0.7 *	13.8 ± 0.7	9.7 ± 0.8 *	0.17 ± 0.01	0.11 ± 0.02 *
HA	79.4 ± 1.9	15.6 ± 2.3	4.6 ± 0.1	0.16 ± 0.03	0.04 ± 0.01 ***
SMPB	84.5 ± 0.8	14.3 ± 1.1	1.2 ± 0.3 ***	0.17 ± 0.01	0.014 ± 0.003 ***
NAC	74.9 ± 4.2 ***	16.7 ± 2.6 *	2.9 ± 0.61 *	0.21 ± 0.04	0.04 ± 0.02 ***

dECM and aECM refer to pristine and plasma-activated ECM, respectively; HA, SMPB, and NAC refer to aECM functionalized with HA, SMPB, or SMPB-NAC, respectively. Results are reported as data mean (*n* = 3), ±standard deviation. *, *** are referred to as statistically significant differences compared to the ECM control, ANOVA–Fisher (*p* < 0.05, 0.001, respectively).

## Data Availability

The raw data supporting the conclusions of this article will be made available by the authors on request.

## Supplementary Materials

The following supporting information can be downloaded at: https://www.mdpi.com/article/10.3390/polym18080977/s1, Figure S1: Metabolic activity produced by the gingival keratinocytes stimulated by different concentrations of hyaluronic acid and/or N-acetyl-L-cysteine; Figure S2: High-resolution XPS O1s analysis of the treated dECM samples, where the spectra were deconvoluted into two main peaks; Figure S3: Schematic representation of the process that may alter the hyaluronic acid pathway to stimulate cell response.

## References

[1] Fitzpatrick S.G., Cohen D.M., Clark A.N. (2019). Ulcerated Lesions of the Oral Mucosa: Clinical and Histologic Review. Head Neck Pathol..

[2] Zhang W., Zhao J., Zou X., Yu J., Liao J., Huang F. (2025). Multifunctional hydrogels for the healing of oral ulcers. J. Biomed. Mater. Res. Part A.

[3] Basso F.G., Pansani T.N., Turrioni A.P., Soares D.G., de Souza Costa C.A., Hebling J. (2016). Tumor Necrosis Factor-α and Interleukin (IL)-1β, IL-6, and IL-8 Impair In Vitro Migration and Induce Apoptosis of Gingival Fibroblasts and Epithelial Cells, Delaying Wound Healing. J. Periodontol..

[4] Hirano T. (2021). IL-6 in inflammation, autoimmunity and cancer. Int. Immunol..

[5] Matsushima K., Yang D., Oppenheim J.J. (2022). Interleukin-8: An evolving chemokine. Cytokine.

[6] Pan Z., Zhang X., Xie W., Cui J., Wang Y., Zhang B., Du L., Zhai W., Sun H., Li Y. (2024). Revisited and innovative perspectives of oral ulcer: From biological specificity to local treatment. Front. Bioeng. Biotechnol..

[7] Vigneswaran N., Muller S., Jeske A.H. (2024). Pharmacologic Treatment of Common Oral Mucosal Inflammatory and Ulcerative Diseases. Contemporary Dental Pharmacology: Evidence-Based Considerations.

[8] Xiang Y., Ren X., Xu Y., Cheng L., Cai H., Hu T. (2023). Anti-Inflammatory and Anti-Bacterial Effects of Mouthwashes in Intensive Care Units: A Systematic Review and Meta-Analysis. Int. J. Environ. Res. Public Health.

[9] Naja J.R., Desparois L., Hebert E.M., Nader M.E.F., Saavedra L., Minahk C.J., Houde V.P. (2025). In vitro modulation of proinflammatory and proteolytic activities of Porphyromonas gingivalis by selected lactobacilli. J. Oral Microbiol..

[10] Singh M., Kim A., Young A., Nguyen D., Monroe C.L., Ding T., Gray D., Venketaraman V. (2024). The Mechanism and Inflammatory Markers Involved in the Potential Use of N-acetylcysteine in Chronic Pain Management. Life.

[11] Riihilä P., Nissinen L., Kähäri V.-M. (2021). Matrix metalloproteinases in keratinocyte carcinomas. Exp. Dermatol..

[12] Luchian I., Goriuc A., Sandu D., Covasa M. (2022). The Role of Matrix Metalloproteinases (MMP-8, MMP-9, MMP-13) in Periodontal and Peri-Implant Pathological Processes. Int. J. Mol. Sci..

[13] Gupta R.C., Lall R., Srivastava A., Sinha A. (2019). Hyaluronic Acid: Molecular Mechanisms and Therapeutic Trajectory. Front. Vet. Sci..

[14] Marinho A., Nunes C., Reis S. (2021). Hyaluronic Acid: A Key Ingredient in the Therapy of Inflammation. Biomolecules.

[15] Fallacara A., Baldini E., Manfredini S., Vertuani S. (2018). Hyaluronic Acid in the Third Millennium. Polymers.

[16] Chrostek L., Cylwik B. (2025). Hyaluronic Acid in Immune Response. Biomolecules.

[17] Lee-Sayer S.S.M., Baldini E., Manfredini S., Vertuani S. (2015). The Where, When, How, and Why of Hyaluronan Binding by Immune Cells. Front. Immunol..

[18] Rosales P., Vitale D., Icardi A., Sevic I., Alaniz L. (2024). Role of Hyaluronic acid and its chemical derivatives in immunity during homeostasis, cancer and tissue regeneration. Semin. Immunopathol..

[19] Ferreira N.d.R., Sanz C.K., Raybolt A., Pereira C.M., DosSantos M.F. (2022). Action of Hyaluronic Acid as a Damage-Associated Molecular Pattern Molecule and Its Function on the Treatment of Temporomandibular Disorders. Front. Pain Res..

[20] Tenório M., Graciliano N.G., Moura F., de Oliveira A.C.M., Goulart M.O.F. (2021). N-Acetylcysteine (NAC): Impacts on Human Health. Antioxidants.

[21] Santus P., Signorello J.C., Danzo F., Lazzaroni G., Saad M., Radovanovic D. (2024). Anti-Inflammatory and Anti-Oxidant Properties of N-Acetylcysteine: A Fresh Perspective. J. Clin. Med..

[22] Kalyanaraman B., Nac N.A.C. (2022). Knockin’ on Heaven’s door: Interpreting the mechanism of action of N-acetylcysteine in tumor and immune cells. Redox Biol..

[23] Ukaegbu K., Allen E., Svoboda K.K.H. (2025). Reactive Oxygen Species and Antioxidants in Wound Healing: Mechanisms and Therapeutic Potential. Int. Wound J..

[24] Pei Y., Liu H., Yang Y., Yang Y., Jiao Y., Tay F.R., Chen J. (2018). Biological Activities and Potential Oral Applications of N-Acetylcysteine: Progress and Prospects. Oxidative Med. Cell. Longev..

[25] Matsuura T., Komatsu K., Ogawa T. (2022). N-Acetyl Cysteine-Mediated Improvements in Dental Restorative Material Biocompatibility. Int. J. Mol. Sci..

[26] Muñoz-González P.U., Lona-Ramos M.C., Gutiérrez-Verdín L.D., Luévano-Colmenero G.H., Tenorio-Rocha F., García-Contreras R., González-García G., la Torre A.R.-D., Delgado J., E Castellano L. (2022). Gel dressing based on type I collagen modified with oligourethane and silica for skin wound healing. Biomed. Mater..

[27] Zhu L., Yu Z.-L., Li S., Xu C.-Z., Hou Y.-J., Liao L.-X., Xu Y.-L., Zhang J.-T., Wei B.-M., Wen W. (2024). Recent Advances on Collagen Biomaterial: From Extraction, Cross-Linking to Tissue Regeneration. Polym. Rev..

[28] Muñoz-González P.U., Delgado J., González-García G., Mendoza-Novelo B. (2025). Stimulation of macrophage cell lines confined with silica and/or silicon particles and embedded in structured collagen gels. J. Biomater. Appl..

[29] Sankar D., Mony U., Rangasamy J. (2021). Combinatorial effect of plasma treatment, fiber alignment and fiber scale of poly (ε-caprolactone)/collagen multiscale fibers in inducing tenogenesis in non-tenogenic media. Mater. Sci. Eng. C.

[30] Firoozi M., Entezam M., Masaeli E., Ejeian F., Nasr-Esfahani M.H. (2022). Physical modification approaches to enhance cell supporting potential of poly (vinyl alcohol)-based hydrogels. J. Appl. Polym. Sci..

[31] Lombardo M.E., Mariscotti V., Chevallier P., Copes F., Boccafoschi F., Sarkissian A., Mantovani D. (2024). Effects of cold plasma treatment on the biological performances of decellularized bovine pericardium extracellular matrix-based films for biomedical applications. Explor. BioMat-X.

[32] Roger P., Delettre J., Bouix M., Béal C. (2011). Characterization of Streptococcus salivarius growth and maintenance in artificial saliva. J. Appl. Microbiol..

[33] Truica-Marasescu F., Wertheimer M.R. (2008). Nitrogen-Rich Plasma-Polymer Films for Biomedical Applications. Plasma Process. Polym..

[34] Bhatt P., Kumar V., Subramaniyan V., Nagarajan K., Sekar M., Chinni S.V., Ramachawolran G. (2023). Plasma Modification Techniques for Natural Polymer-Based Drug Delivery Systems. Pharmaceutics.

[35] Mohammadi X., Matinfar G., Hossain A., Pratap-Singh A. (2026). Cold-plasma surface engineering induces multifunctional enhancements in biodegradable packaging films. Trends Food Sci. Technol..

[36] Chytrosz-Wrobel P., Golda-Cepa M., Stodolak-Zych E., Rysz J., Kotarba A. (2023). Effect of oxygen plasma-treatment on surface functional groups, wettability, and nanotopography features of medically relevant polymers with various crystallinities. Appl. Surf. Sci. Adv..

[37] Xiao H., Wu M., Shao F., Guan G., Huang B., Tan B., Yin Y. (2016). N-Acetyl-L-cysteine Protects the Enterocyte against Oxidative Damage by Modulation of Mitochondrial Function. Mediat. Inflamm..

[38] Sahasrabudhe S.A., Terluk M.R., Kartha R.V. (2023). N-acetylcysteine Pharmacology and Applications in Rare Diseases—Repurposing an Old Antioxidant. Antioxidants.

[39] Hu L., Nomura S., Sato Y., Takagi K., Ishii T., Honma Y., Watanabe K., Mizukami Y., Muto J. (2022). Anti-inflammatory effects of differential molecular weight Hyaluronic acids on UVB-induced calprotectin-mediated keratinocyte inflammation. J. Dermatol. Sci..

[40] Chen M., Li L., Wang Z., Li P., Feng F., Zheng X. (2019). High molecular weight hyaluronic acid regulates P. gingivalis–induced inflammation and migration in human gingival fibroblasts via MAPK and NF-κB signaling pathway. Arch. Oral Biol..

[41] Groeger S.E., Meyle J. (2015). Epithelial barrier and oral bacterial infection. Periodontology 2000.

[42] Olsson-Brown A., Yip V., Ogiji E.D., Jolly C., Ressel L., Sharma A., Bergfeld W., Liu X., Khirwadkar N., Bellon T. (2023). TNF-α—Mediated Keratinocyte Expression and Release of Matrix Metalloproteinase 9: Putative Mechanism of Pathogenesis in Stevens—Johnson Syndrome/Toxic Epidermal Necrolysis. J. Investig. Dermatol..

