# Supplementary material for: A Plasma-Functionalized ECM Platform for Intraoral Inflammation Control: Comparative Effects of Hyaluronic Acid and N-Acetyl-L-Cysteine on Oral Keratinocyte Response
Source: Polymers (Basel). 2026 Apr 17;18(8):977. doi: 10.3390/polym18080977 (PMC13120145; doi:10.3390/polym18080977)
Supplement: Supplementary file 1 [file polymers-18-00977-s001.zip › polymers-4199868-supplementary.pdf]

Supplementary information of A Plasma-Functionalized ECM Platform for Intraoral Inflammation Control: Comparative Effects of Hyaluronic Acid and N-acetyl-L-cysteine on Oral Keratinocyte Response.

Muñoz-González Pedro U.[‡], Chevallier Pascale[‡], Desparois Leyla[□], Avon Sylvie Louise[#], Chandad Fatiha[□], Mantovani Diego[‡], Houde Vanessa P.[□,]*

[‡] Laboratory for Biomaterials and Bioengineering (LBB), Laval University, Québec, Quebec G1V 0A6, Canada

[□]Oral Ecology Research Group (GREB), Faculty of Dental Medicine, Université Laval, Québec, QC, Canada

[#]Faculty of Dental Medicine, Université Laval, Québec, QC, Canada

**vanessa.houde@fmd.ulaval.ca*

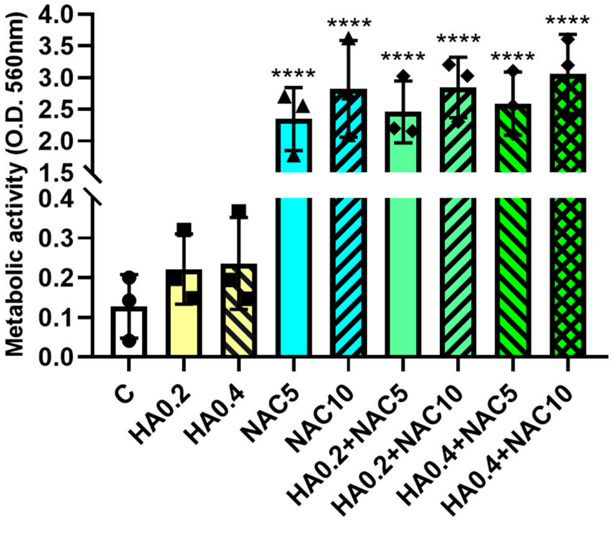

Fig. S1: Metabolic activity produced by the gingival keratinocytes stimulated by different concentrations of hyaluronic acid and/or N-acetyl-L-cysteine. **Legend:** C refers to unstimulated keratinocytes control, HA 0.4 and 0.2 refer to keratinocytes stimulated with 0.4 and 0.2% hyaluronic acid, respectively; NAC10 and 5 refer to keratinocytes stimulated with 10 and 5mM N-acetyl-L-cysteine, respectively. Results are reported as data overlap, mean (large bar n=3), ± standard deviation (short bars). *, **, ***, **** are referred to as statistically significant differences compared to the control ANOVA-Fisher ($p < 0.05, 0.01, 0.001, 0.0001$, respectively).

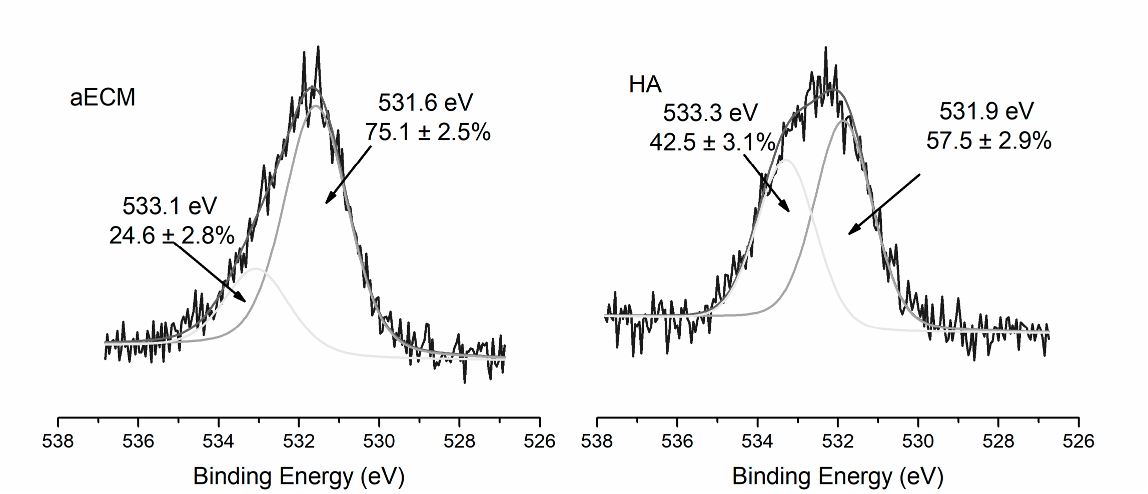

Fig. S2: High-resolution XPS O1s analysis of the treated dECM samples, where the spectra were deconvoluted into two main peaks. **Legend:** aECM refers to decellularized extracellular matrix plasma-activated; HA refers to aECM functionalized with hyaluronic acid.

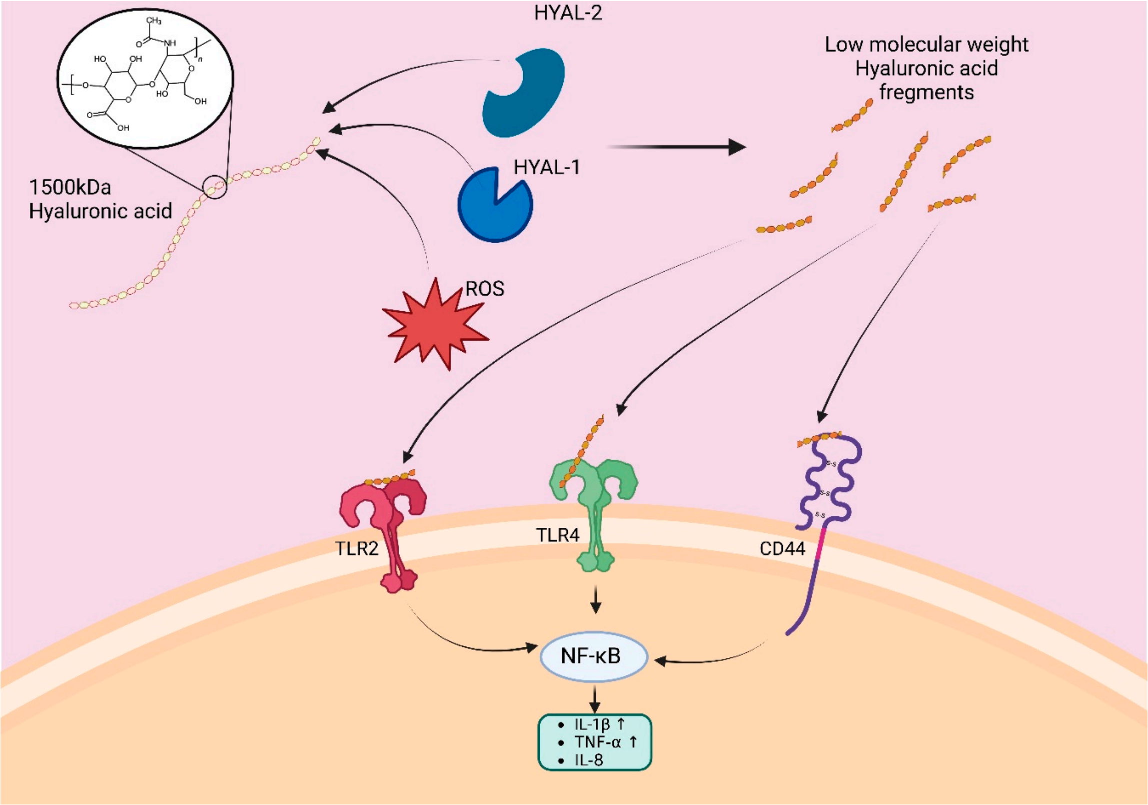

Fig. S3: Schematic representation of the process that may alter the hyaluronic acid pathway to stimulate cell response, where it can be seen that the original 1500kDa hyaluronic acid molecule may be degraded by HYAL-1, HYAL-2, or ROS, to produce low molecular weight fragments that promote the production of pro-inflammatory cytokines via the activation of NF-κB pathway through TLR2, TLR4, and CD44 receptors.
